# Administration of chiglitazar reverses chronic stress-induced depressive-like symptoms in mice via activation of hippocampal PPARα and BDNF

**DOI:** 10.3389/fphar.2025.1587399

**Published:** 2025-04-28

**Authors:** Jiu-Jian Zhou, Jie Zhao, Shang-Yan Gao, Yuan-yuan Gao, Cheng Chen, Yi Ding, Zhong-hua Wu, Pu-Jian Chen

**Affiliations:** ^1^ Department of Emergency, The Affiliated Nantong Hospital of Shanghai University (The Sixth People’s Hospital of Nantong), Nantong, Jiangsu, China; ^2^ Department of Clinical Pharmacy, The Affiliated Nantong Hospital of Shanghai University (The Sixth People’s Hospital of Nantong), Nantong, Jiangsu, China; ^3^ Department of Neurology, The Affiliated Nantong Hospital of Shanghai University (The Sixth People’s Hospital of Nantong), Nantong, Jiangsu, China; ^4^ Department of Neurosurgery, The Affiliated Nantong Hospital of Shanghai University (The Sixth People’s Hospital of Nantong), Nantong, Jiangsu, China; ^5^ Department of General Practice, The Affiliated Nantong Hospital of Shanghai University (The Sixth People’s Hospital of Nantong), Nantong, Jiangsu, China

**Keywords:** brain-derived neurotrophic factor, chiglitazar, chronic stress, depression, hippocampus, peroxisome proliferator-activated receptor α

## Abstract

**Background:**

Developing non-monoamine based novel antidepressants is now popular and necessary. Peroxisome proliferator-activated receptor α (PPARα) has been demonstrated to play a role in the pathophysiology of depression, and several PPARα agonists including WY14643, fenofibrate, and gemfibrozil, have all been reported to possess antidepressant-like efficacy in rodents. Chiglitazar is a novel pan agonist of PPARs, and this study aims to investigate whether this agonist has beneficial effects against depression.

**Methods:**

Chronic unpredictable mild stress (CUMS), chronic restraint stress (CRS), forced swim test (FST), tail suspension test (TST), sucrose preference test (SPT), western blotting, and adeno-associated virus (AAV)-mediated gene transfer were adopted together in the present study.

**Results:**

It was found that repeated intraperitoneal (i.p.) injection of chiglitazar significantly reversed both CUMS-induced and CRS-induced depressive-like behaviors in mice in the FST, TST, and SPT. Chiglitazar treatment also fully reversed both CUMS-induced and CRS-induced downregulation in the expression of hippocampal PPARα and brain-derived neurotrophic factor (BDNF) signaling in mice. Furthermore, pharmacological blockade of hippocampal PPARα and BDNF signaling attenuated the antidepressant-like effects of chiglitazar in mice. Genetic knockdown of hippocampal PPARα and BDNF also abolished the antidepressant-like actions of chiglitazar in mice.

**Conclusion:**

In summary, administration of chiglitazar reverses chronic stress-induced depressive-like symptoms in mice via activation of hippocampal PPARα and BDNF.

## Highlights

• Chiglitazar treatment reversed both CUMS-induced and CRS-induced depressive-like behaviors in mice.
• Chiglitazar treatment reversed both CUMS-induced and CRS-induced decrease in hippocampal PPARα and BDNF signaling in mice.
• Pharmacological blockade of hippocampal PPARα and BDNF attenuated the antidepressant-like effects of chiglitazar in mice.
• Genetic knockdown of hippocampal PPARα and BDNF abolished the antidepressant-like actions of chiglitazar in mice.

## Introduction

As a most serious psychiatric disorder which threats about one-sixth of the population in this world in 21st century, depression causes huge costs to both families and society (Simon et al., 2024). The monoamine hypothesis of depression has been the most widely acknowledged in the past few decades and up to date, nearly all commercial antidepressants used in clinical practice are developed according to this etiology Dean and Keshavan, 2017; Perez-Caballero et al., 2019). However, it has been found that for selective serotonin reuptake inhibitors (SSRIs) and serotonin and noradrenaline reuptake inhibitors (SNRIs), less than 50% of patients are effective, and moreover, weeks of administration are always needed to produce therapeutic efficacy (Blier and El Mansari, 2013; Gonda et al., 2023). Besides, SSRIs and SNRIs have various side effects (Blier and El Mansari, 2013; Gonda et al., 2023). Therefore, developing non-monoamine based novel antidepressants is now popular and necessary.

As a well-known neurotrophic factor, brain-derived neurotrophic factor (BDNF) is widely distributed throughout the brain and plays a key role in neurogenesis, neuro-survival, and synaptic plasticity (Greenberg et al., 2009; Leal et al., 2014; Liu and Nusslock, 2018). It has been well-demonstrated that the BDNF signaling cascade mainly include BDNF, tyrosine kinase B (TrkB), extracellular regulated protein kinase (ERK), protein kinase B (AKT), and cAMP response element-binding protein (CREB) (Sasi et al., 2017; Kandezi et al., 2020; Zarneshan et al., 2022). BDNF induces ser-133 phosphorylation of nuclear CREB by binding membrane TrkB receptor and then activating cytoplasmic ERK and AKT (Sasi et al., 2017; Kandezi et al., 2020; Zarneshan et al., 2022). Many previous reports have shown that the BDNF signaling cascade is closely involved in the pathophysiology of depression (Björkholm and Monteggia, 2016). For example, Filho *et al.* indicated that BDNF expression is significantly downregulated in the hippocampus of mice exposed to chronic stress (Filho et al., 2015). MacQueen *et al.* indicated that compared with wild-type mice, heterogeneous BDNF knockout mice displayed notable depressive-like behaviors (MacQueen et al., 2001). Mendez-David *et al.* showed that the BDNF system participates in the antidepressant mechanism of fluoxetine, a most well-known SSRI (Mendez-David et al., 2015). Tikhonova *et al.* showed that administration of exogeneous BDNF protein into the hippocampus induced antidepressant-like actions in rodents (Tikhonova and Kulikov, 2012). As to how BDNF correlates with depression, currently it is widely accepted that changes in BDNF expression reverses or exacerbates depression-associated dysfunction in neurogenesis, neuro-survival, and synaptic plasticity, since BDNF promotes the survival of existing neurons and encourages the growth and differentiation of new neurons and synapses (Dean and Keshavan, 2017)

Chiglitazar is a novel pan agonist of peroxisome proliferator-activated receptors (PPARs) and approved in China in October 2021 for the treatment of type 2 diabetes and non-alcoholic steatohepatitis (DeFronzo, 2021; Deeks, 2022). PPARs have three members: PPARα, PPARδ, and PPARγ (Titus et al., 2024). Interestingly, PPARα is closely implicated in the pathophysiology of depression (Scheggi et al., 2016; Chen et al., 2019a; Wang et al., 2021b; Gao S. et al., 2022), and in 2018, Song *et al.* explored the role of central PPARα in depression comprehensively (Song et al., 2018). It has been found that chronic stress notably downregulated both PPARα expression and PPARα-CREB binding in the hippocampus (Song et al., 2018). Hippocampal PPARα overexpression produced notable antidepressant-like effects in mice by promoting CREB-mediated BDNF biosynthesis (Song et al., 2018). In contrast, both knockdown and knockout of PPARα aggravated depression in mice (Song et al., 2018). Moreover, hippocampal PPARα participated in the antidepressant mechanism of fluoxetine (Song et al., 2018). In addition, several other PPARα agonists, WY14643, fenofibrate, and gemfibrozil, have all been reported to possess antidepressant-like actions in mice via promoting hippocampal PPARα and BDNF (Jiang et al., 2015; Jiang et al., 2017; Ni et al., 2018). Thus, here we assumed that chiglitazar may be another antidepressant candidate, and in the present study, various methods were adopted to investigate this possibility.

## Materials and methods

### Chronic unpredictable mild stress (CUMS)

The experimental approach for the CUMS model has been frequently described in previous reports (Liu et al., 2020; Liu et al., 2021; Zhao et al., 2021; Wu et al., 2022; Huang et al., 2023) and involves eight different stressors: 1, 2 h of restraint; 2, light/dark cycle inversion; 3, 12 h of exposure to damp sawdust; 4, 1 h of cold stress at 4 °C; 5, 12 h of cage tilting (45 °C) in an empty cage; 6, 30 min of cage rotation; 7, 23 h of food deprivation; and 8, 23 h of food deprivation. All C57BL/6J mice in the stressed groups individually underwent an 8-week period of CUMS, and for each week, the ranking of stressors was randomly assigned. Administration of drugs were performed daily during the last 2 weeks. In contrast, for the non-stressed groups, C57BL/6J mice were handled daily. The forced swim test (FST; First), tail suspension test (TST; Second), and sucrose preference test (SPT; Third) were adopted for behavioral assays.

### Chronic restraint stress (CRS)

This model of depression was done as mentioned in several previous studies (Chen et al., 2019a; Wu et al., 2022). Briefly, mice in the stressed groups were individually subjected to 8 weeks of CRS (3 h/d, 9:00 a.m. to 12:00 a.m.), and administration of drugs was performed daily in the last 2 weeks. Conical plastic tubes (containing vent holes) of 50 ml were used for restraint stress. Mice in the control group were handled daily. The depressive-like behaviors of mice were assayed using the FST (First), TST (Second) and SPT (Third).

### Additional methods and materials

See the Supplemental Methods and Materials for description of animals, materials, FST, TST, SPT, open field test (OFT), adeno-associated virus (AAV)-mediated gene transfer, western blotting, statistics, and other details.

## Results

### Single injection of chiglitazar exhibited antidepressant-like potential in the FST and TST without affecting mice locomotor activity

**FIGURE 1:**
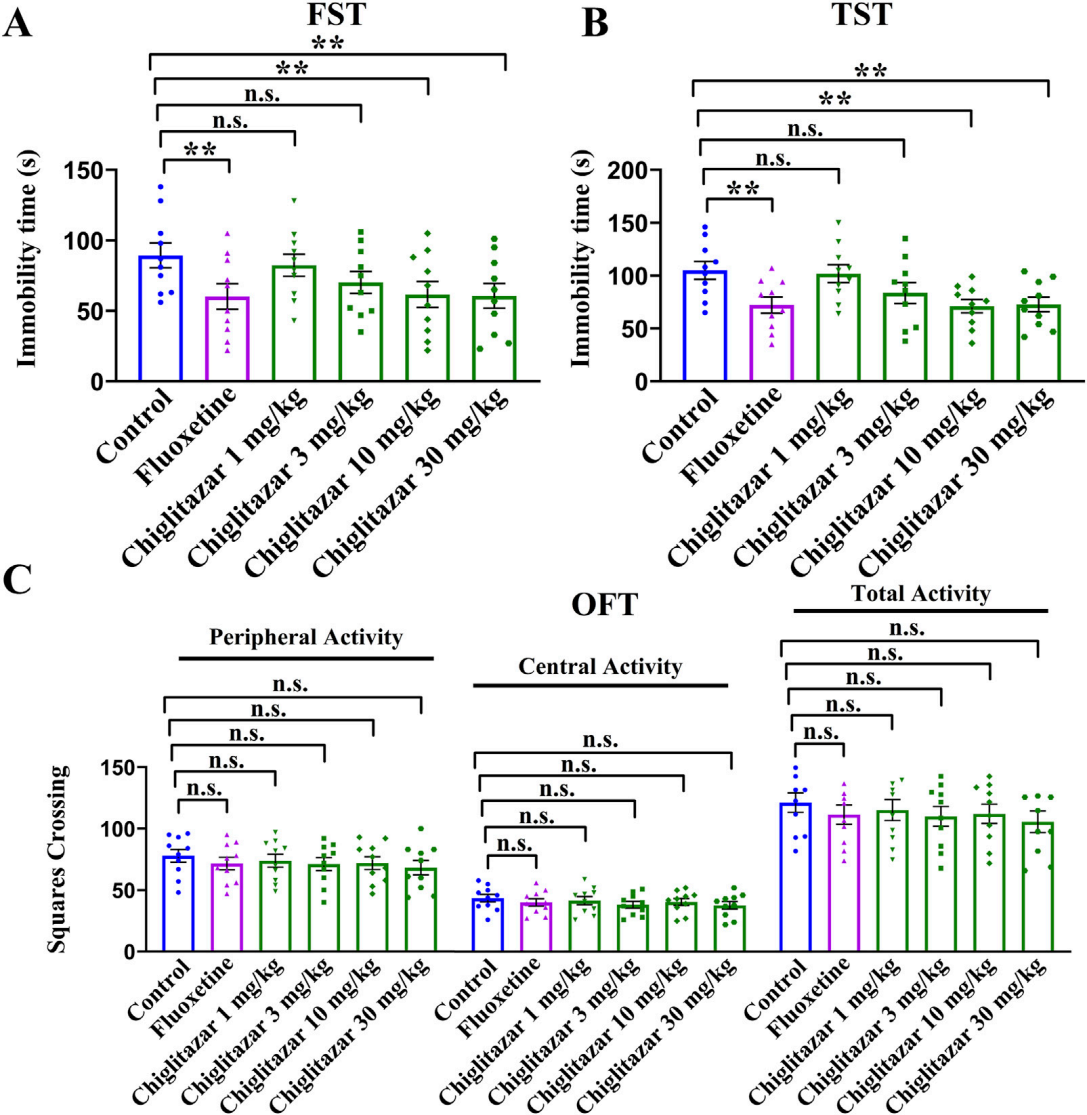
Naïve C57BL/6J mice received a single i.p. injection of fluoxetine (20 mg/kg), chiglitazar (1, 3, 10, and 30 mg/kg) or vehicle, followed by the FST, TST or OFT (after 30 min). **(A, B)** Administration of 10 and 30 mg/kg chiglitazar significantly reduced the immobility duration of naïve mice in both the FST and TST, producing an effect similar to that of 20 mg/kg fluoxetine, whereas treatment of 1 mg/kg and 3 mg/kg chiglitazar did not induce such behavioral changes. **(C)** Neither chiglitazar nor fluoxetine produced significant influence on the locomotor activity of naive mice in the OFT, as there is no significant differences among all groups in the number of squares that a mouse crossed in the central or peripheral area. All data were represented as means ± S.E.M (n = 10); **P < 0.01; n.s., no significance. The comparisons were made by one-way ANOVA followed by Tukey’s test.

As the first step of this study, chiglitazar was examined for whether possessing antidepressant-like potential using the FST, TST, OFT, and naïve C57BL/6J mice. As shown in Figure 1A, B, a single intraperitoneal (i.p.) injection of the positive control, 20 mg/kg fluoxetine, evidently reduced the immobility duration of naïve mice in the FST and TST by 32.6% ± 4.51% and 31.2% ± 3.87%, respectively (n = 10, *P* < 0.01). A single i.p. injection of 10 mg/kg chiglitazar achieved similar efficacy to 20 mg/kg fluoxetine (n = 10, *P* < 0.01), whereas injection of 1 and 3 mg/kg chiglitazar induced non-significant effects (n = 10). Moreover, the effects of 30 mg/kg chiglitazar were comparable with, but not superior to those of 10 mg/kg chiglitazar (n = 10, *P* < 0.01). For the FST data, one-way ANOVA indicates a notable effect of drug treatment [F(5, 54) = 14.625, *P* < 0.01]. For the TST data, one-way ANOVA also reveals a notable effect of drug treatment [F(5, 54) = 17.039, *P* < 0.01].

Figure 1C demonstrated that a single i.p. injection of both fluoxetine and chiglitazar produced none influence on the locomotor activity of naïve mice (n = 10), excluding the possibility that the behavioral changes in Figure 1A, B may attribute to enhanced locomotor activity of animals. One-way ANOVA shows no effects of drug treatment [F(5, 54) = 1.534, *P* > 0.05]. Therefore, 10 mg/kg was chosen as the dose for chiglitazar in the following studies.

### Repeated administration of chiglitazar fully reversed both CUMS-induced and CRS-induced depressive-like behaviors in mice

**FIGURE 2:**
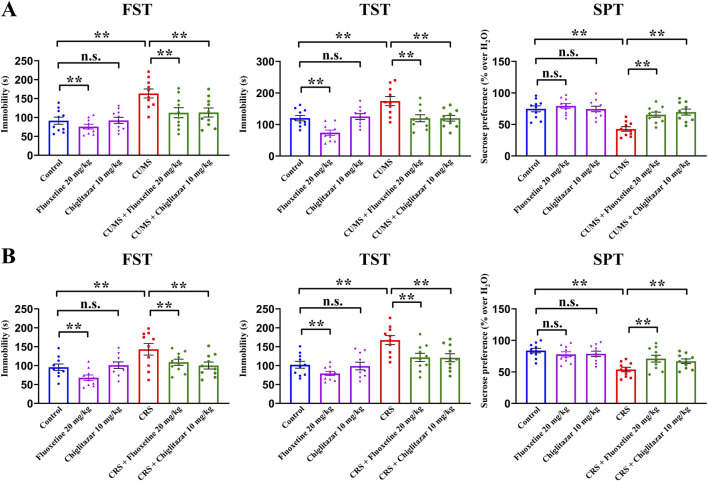
Adult C57BL/6J mice received 8 weeks of CUMS or CRS, and i.p. injection of 20 mg/kg fluoxetine, 10 mg/kg chiglitazar or vehicle was performed daily during the last 2 weeks. **(A)** It was found that repeated injection of both 20 mg/kg fluoxetine and 10 mg/kg chiglitazar significantly reversed the CUMS-induced increase in mice immobility in the FST and TST as well as decrease in mice sucrose preference in the SPT. **(B)** It was found that repeated injection of both 20 mg/kg fluoxetine and 10 mg/kg chiglitazar also notably reversed the CRS-induced increase in mice immobility in the FST andTST as well as decrease in mice sucrose preference in the SPT. All data were represented as means ± S.E.M (n = 10); **P < 0.01; n.s., no significance. The comparisons were made by two-way ANOVA followed by Bonferroni’s test.

The antidepressant-like effects of chiglitazar were then evaluated using both the CUMS and CRS models of depression. Figure 2A summarizes the behavioral results for the CUMS-involved experiments. Compared with mice in the control group, CUMS exposure significantly increased mice immobility in the FST and TST by 78.8% ± 9.25% and 44.8% ± 6.16%, respectively, and notably decreased the sucrose preference of mice by 42.8% ± 5.37% (n = 10, *P* < 0.01). In contrast, repeated administration of 10 mg/kg chiglitazar reduced the immobility of CUMS-treated mice in the FST and TST by 30.8% ± 5.41% and 31.2% ± 4.69%, respectively, and enhanced the sucrose preference of CUMS-treated mice by 62.9% ± 8.27% (n = 10, *P* < 0.01). Repeated administration of the positive control, 20 mg/kg fluoxetine, achieved similar efficacy (n = 10, *P* < 0.01). Fluoxetine treatment also decreased the immobility of naïve control mice in the FST and TST, whereas chiglitazar treatment did not (n = 10, *P* < 0.01). For the FST data, two-way ANOVA reveals a significant interaction [F(2, 54) = 18.229, *P* < 0.01] with notable effects for CUMS [F(1, 54) = 31.845, *P* < 0.01] and drug treatment [F(2, 54) = 24.781, *P* < 0.01]. For the TST data, two-way ANOVA shows a significant interaction [F(2, 54) = 15.117, *P* < 0.01] with notable effects for CUMS [F(1, 54) = 26.347, *P* < 0.01] and drug treatment [F(2, 54) = 19.501, *P* < 0.01]. For the SPT data, two-way ANOVA also reports a significant interaction [F(2, 54) = 14.609, *P* < 0.01] with notable effects for CUMS [F(1, 54) = 27.586, *P* < 0.01] and drug treatment [F(2, 54) = 20.144, *P* < 0.01].

Figure 2B summarizes the behavioral results for the CRS-involved experiments. As well as CUMS, compared with mice in the control group, CRS exposure increased mice immobility in the FST and TST by 49.5% ± 5.15% and 63.9% ± 8.04%, respectively, and decreased the sucrose preference of mice by 35.8% ± 4.72% (n = 10, *P* < 0.01). Repeated treatment of both 10 mg/kg chiglitazar and 20 mg/kg fluoxetine fully reversed all CRS-induced behavioral changes in the FST, TST, and SPT (n = 10, *P* < 0.01). For the FST data, two-way ANOVA reveals a significant interaction [F(2, 54) = 17.236, *P* < 0.01] with notable effects for CUMS [F(1, 54) = 25.123, *P* < 0.01] and drug treatment [F(2, 54) = 21.549, *P* < 0.01]. For the TST data, two-way ANOVA shows a significant interaction [F(2, 54) = 16.075, *P* < 0.01] with notable effects for CUMS [F(1, 54) = 28.139, *P* < 0.01] and drug treatment [F(2, 54) = 22.824, *P* < 0.01]. For the SPT data, two-way ANOVA also reports a significant interaction [F(2, 54) = 12.551, *P* < 0.01] with notable effects for CUMS [F(1, 54) = 22.614, *P* < 0.01] and drug treatment [F(2, 54) = 16.268, *P* < 0.01].

Taken together, these results suggest that chiglitazar possesses potential of being an antidepressant.

Repeated chiglitazar treatment significantly reversed both CUMS-induced and CRS-induced inhibitory effects on hippocampal PPARα and BDNF signaling in mice.

After the behavioral tests, western blotting was performed to detect the protein expression of PPARα and the whole BDNF signaling cascade in the hippocampus among all groups of mice.

**FIGURE 3:**
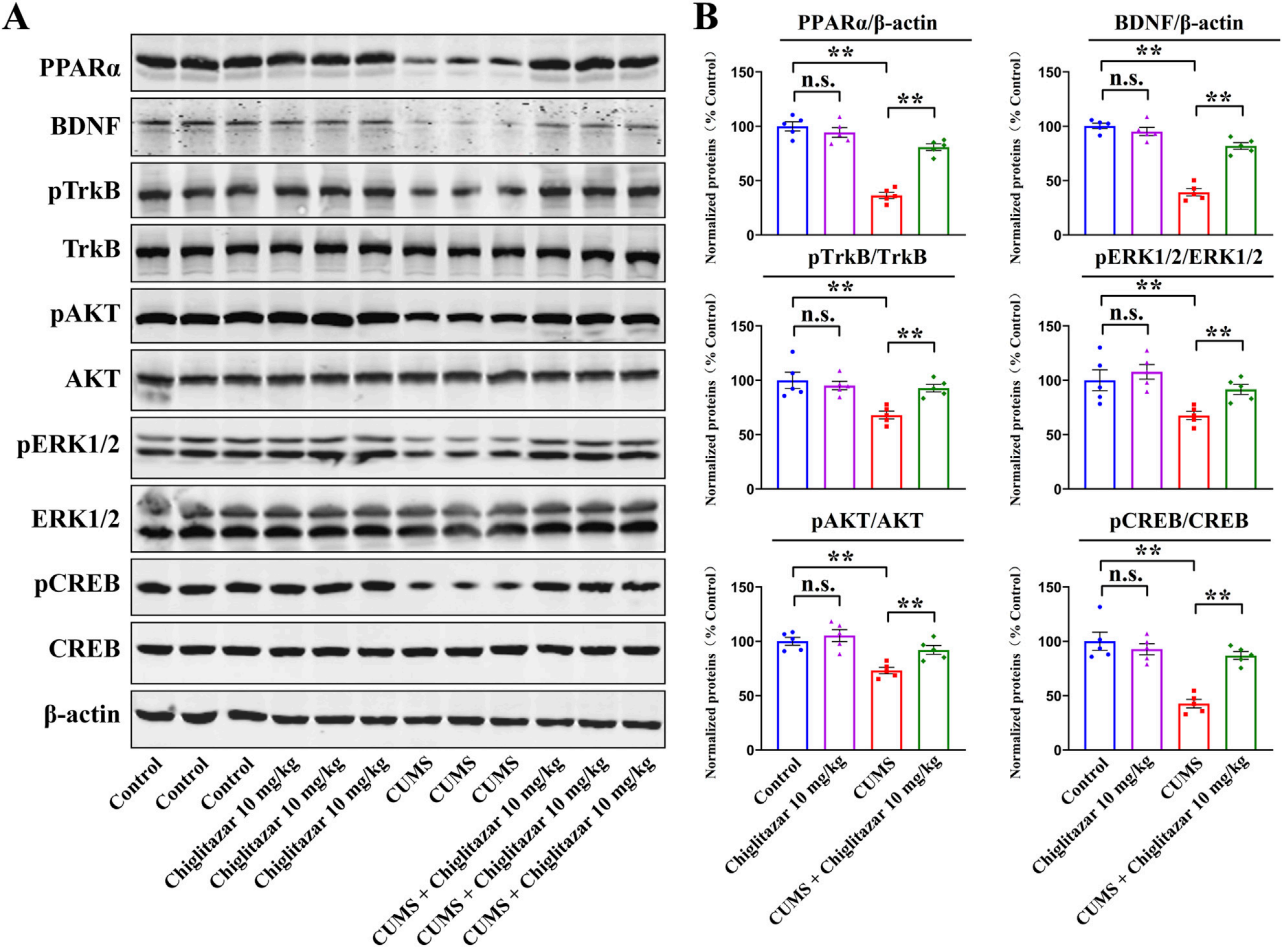
Representative western blotting images **(A)** and corresponding data analyses **(B)** together show the effects of CUMS and 10 mg/kg chiglitazar on the protein expression of PPARα, BDNF, pTrkB, TrkB, pAKT, AKT, pERK1/2, ERK1/2, pCREB, and CREB in the hippocampus of male C57BL/6J mice. It was found that the usage of chiglitazar evidently antagonized the inhibitory effects of CUMS on hippocampal PPARα, BDNF, pTrkB, pAKT, pERK1/2, and pCREB. All data were represented as means ± S.E.M (n = 5); **P < 0.01; n.s., no significance. The comparisons were made by two-way ANOVA followed by Bonferroni’s test.

Figure 3 summarizes the western blotting data for the CUMS-involved experiments. Compared with mice in the control group, CUMS exposure significantly downregulated the protein levels of hippocampal PPARα [ANOVA: CUMS, F(1, 16) = 36.581, *P* < 0.01; Drug treatment, F(1, 16) = 28.177, *P* < 0.01; Interaction, F(1, 16) = 22.549, *P* < 0.01], BDNF [ANOVA: CUMS, F(1, 16) = 34.662, *P* < 0.01; Drug treatment, F(1, 16) = 27.535, *P* < 0.01; Interaction, F(1, 16) = 18.404, *P* < 0.01], pTrkB [ANOVA: CUMS, F(1, 16) = 22.905, *P* < 0.01; Drug treatment, F(1, 16) = 17.876, *P* < 0.01; Interaction, F(1, 16) = 14.112, *P* < 0.01], pAKT [ANOVA: CUMS, F(1, 16) = 20.755, *P* < 0.01; Drug treatment, F(1, 16) = 15.402, *P* < 0.01; Interaction, F(1, 16) = 11.934, *P* < 0.01], pERK1/2 [ANOVA: CUMS, F(1, 16) = 24.672, *P* < 0.01; Drug treatment, F(1, 16) = 18.974, *P* < 0.01; Interaction, F(1, 16) = 14.108, *P* < 0.01], and pCREB [ANOVA: CUMS, F(1, 16) = 35.603, *P* < 0.01; Drug treatment, F(1, 16) = 28.079, *P* < 0.01; Interaction, F(1, 16) = 22.175, *P* < 0.01] in mice (n = 5, *P* < 0.01). In contrast, repeated administration of 10 mg/kg chiglitazar notably upregulated the protein levels of these molecules in CUMS-treated mice (n = 5, *P* < 0.01). The protein levels of total TrkB, AKT, ERK1/2, and CREB remain constant between all groups of mice (n = 5).

**FIGURE 4:**
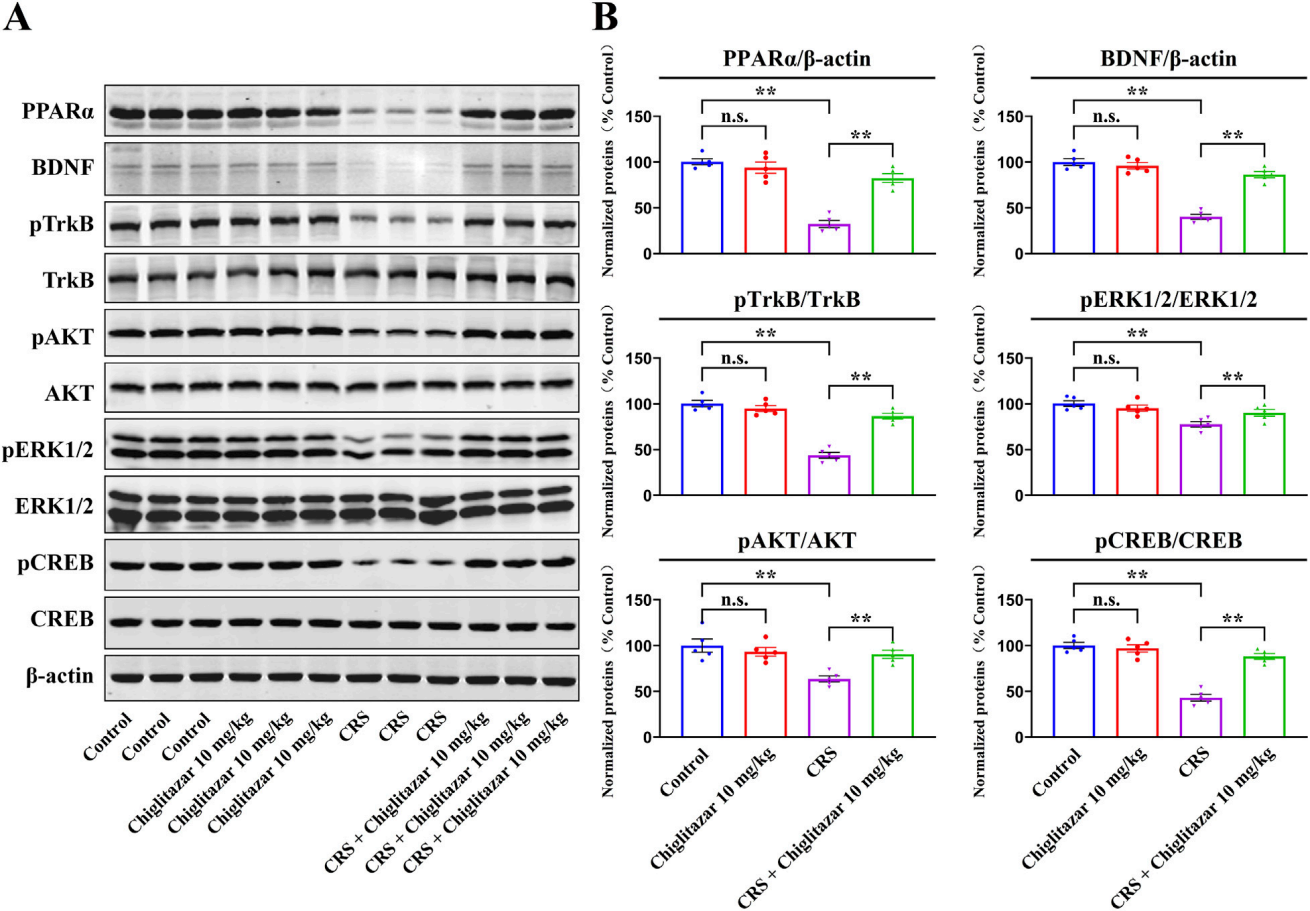
Representative western blotting images **(A)** and corresponding data analyses **(B)** together show the effects of CRS and 10 mg/kg chiglitazar on the protein expression of PPARα, BDNF, pTrkB, TrkB, pAKT, AKT, pERK1/2, ERK1/2, pCREB, and CREB in the hippocampus of male C57BL/6J mice. It was found that the usage of chiglitazar evidently antagonized the inhibitory effects of CUMS on hippocampal PPARα, BDNF, pTrkB, pAKT, pERK1/2, and pCREB. All data were represented as means ± S.E.M (n = 5); **P < 0.01; n.s., no significance. The comparisons were made by two-way ANOVA followed by Bonferroni’s test.

Figure 4 summarizes the western blotting data for the CRS-involved experiments. As well as CUMS, compared with mice in the control group, CRS exposure remarkably downregulated the protein levels of hippocampal PPARα [ANOVA: CRS, F(1, 16) = 42.175, *P* < 0.01; Drug treatment, F(1, 16) = 32.942, *P* < 0.01; Interaction, F(1, 16) = 25.418, *P* < 0.01], BDNF [ANOVA: CRS, F(1, 16) = 38.641, *P* < 0.01; Drug treatment, F(1, 16) = 29.677, *P* < 0.01; Interaction, F(1, 16) = 23.528, *P* < 0.01], pTrkB [ANOVA: CRS, F(1, 16) = 36.199, *P* < 0.01; Drug treatment, F(1, 16) = 28.762, *P* < 0.01; Interaction, F(1, 16) = 20.675, *P* < 0.01], pAKT [ANOVA: CRS, F(1, 16) = 25.488, *P* < 0.01; Drug treatment, F(1, 16) = 19.083, *P* < 0.01; Interaction, F(1, 16) = 15.211, *P* < 0.01], pERK1/2 [ANOVA: CRS, F(1, 16) = 16.337, *P* < 0.01; Drug treatment, F(1, 16) = 13.524, *P* < 0.01; Interaction, F(1, 16) = 10.206, *P* < 0.01], and pCREB [ANOVA: CRS, F(1, 16) = 37.802, *P* < 0.01; Drug treatment, F(1, 16) = 28.965, *P* < 0.01; Interaction, F(1, 16) = 19.256, *P* < 0.01] in mice (n = 5, *P* < 0.01), and all these molecular changes were fully reversed by 10 mg/kg chiglitazar treatment (n = 5, *P* < 0.01). The protein levels of total TrkB, AKT, ERK1/2, and CREB remain constant between all groups of mice (n = 5).

Collectively, these findings indicate that chiglitazar may produce antidepressant-like effects in mice by promoting hippocampal PPARα and BDNF signaling.

### Pharmacological blockade of hippocampal PPARα and BDNF signaling attenuated the antidepressant-like effects of chiglitazar in mice

**FIGURE 5:**
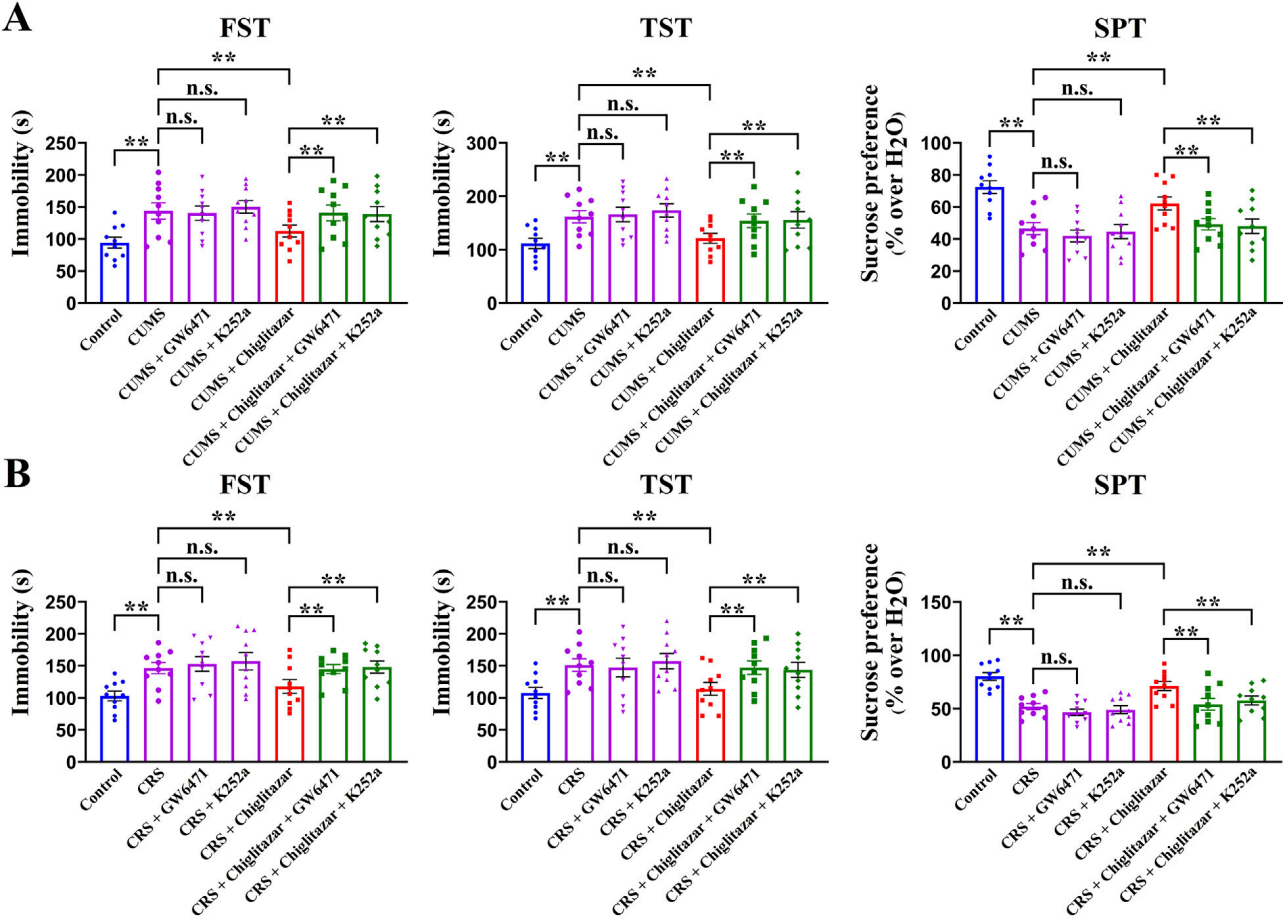
Adult C57BL/6J mice received 8 weeks of CUMS or CRS, and i.p. injection of vehicle, 1 mg/kg GW6471, 25 μg/kg K252a, 10 mg/kg chiglitazar, (10 mg/kg chiglitazar + 1 mg/kg GW6471) or (10 mg/kg chiglitazar + 25 μg/kg K252a) was performed daily during the last 2 weeks. **(A)** It was found that co-administration of both 1 mg/kg GW6471 and 25 μg/kg K252a significantly attenuated the reversal effects of 10 mg/kg chiglitazar against the CUMS-induced increase in mice immobility in the FST and TST as well as decrease in mice sucrose preference in the SPT. **(B)** It was found that co-treatment of both 1 mg/kg GW6471 and 25 μg/kg K252a also notably blocked the reversal effects of 10 mg/kg chiglitazar against the CRS-induced increase in mice immobility in the FST and TST as well as decrease in mice sucrose preference in the SPT. All data were represented as means ± S.E.M (n = 10); **P < 0.01; n.s., no significance. The comparisons were made by one-way ANOVA followed by Tukey’s test.

Furthermore, to determine whether hippocampal PPARα and BDNF signaling are indeed necessary for the antidepressant-like actions of chiglitazar in mice, GW6471 and K252a, two potent antagonists respectively for PPARα and TrkB, were used together. Figure 5A shows that co-administration of GW6471 and K252a significantly attenuated the antidepressant-like effects of chiglitazar in the CUMS model of depression (n = 10, *P* < 0.01). Detailed analyses reveal that the (CUMS + chiglitazar + GW6471)-treated and (CUMS + chiglitazar + K252a)-treated mice respectively displayed 25.3% ± 3.05% and 23.6% ± 4.21% higher immobility in the FST than the (CUMS + chiglitazar)-treated mice [ANOVA: F(6, 63) = 18.785, *P* < 0.01]. The (CUMS + chiglitazar + GW6471)-treated and (CUMS + chiglitazar + K252a)-treated mice also respectively exhibited 26.9% ± 2.45% and 28.3% ± 3.34% higher immobility in the TST than the (CUMS + chiglitazar)-treated mice [ANOVA: F(6, 63) = 23.441, *P* < 0.01]. Moreover, the (CUMS + chiglitazar + GW6471)-treated and (CUMS + chiglitazar + K252a)-treated mice respectively had 20.9% ± 3.73% and 22.8% ± 4.19% lower sucrose preference than the (CUMS + chiglitazar)-treated mice [ANOVA: F(6, 63) = 15.627, *P* < 0.01]. Figure 5B shows that co-treatment with GW6471 and K252a evidently blocked the antidepressant-like actions of chiglitazar in the CRS model of depression (n = 10, *P* < 0.01). Detailed analyses reveal that the (CRS + chiglitazar + GW6471)-treated and (CRS + chiglitazar + K252a)-treated mice respectively displayed 23.1% ± 4.46% and 25.8% ± 3.72% more immobility in the FST than the (CRS + chiglitazar)-treated mice [ANOVA: F(6, 63) = 21.577, *P* < 0.01]. The (CRS + chiglitazar + GW6471)-treated and (CRS + chiglitazar + K252a)-treated mice also respectively exhibited 29.1% ± 3.54% and 26.1% ± 2.39% more immobility in the TST than the (CRS + chiglitazar)-treated mice [ANOVA: F(6, 63) = 26.374, *P* < 0.01]. In addition, the (CRS + chiglitazar + GW6471)-treated and (CRS + chiglitazar + K252a)-treated mice respectively had 24% ± 4.03% and 18.9% ± 3.17% less sucrose preference than the (CRS + chiglitazar)-treated mice [ANOVA: F(6, 63) = 16.329, *P* < 0.01].

### Genetic knockdown of hippocampal PPARα and BDNF abolished the antidepressant-like actions of chiglitazar in mice

**FIGURE 6:**
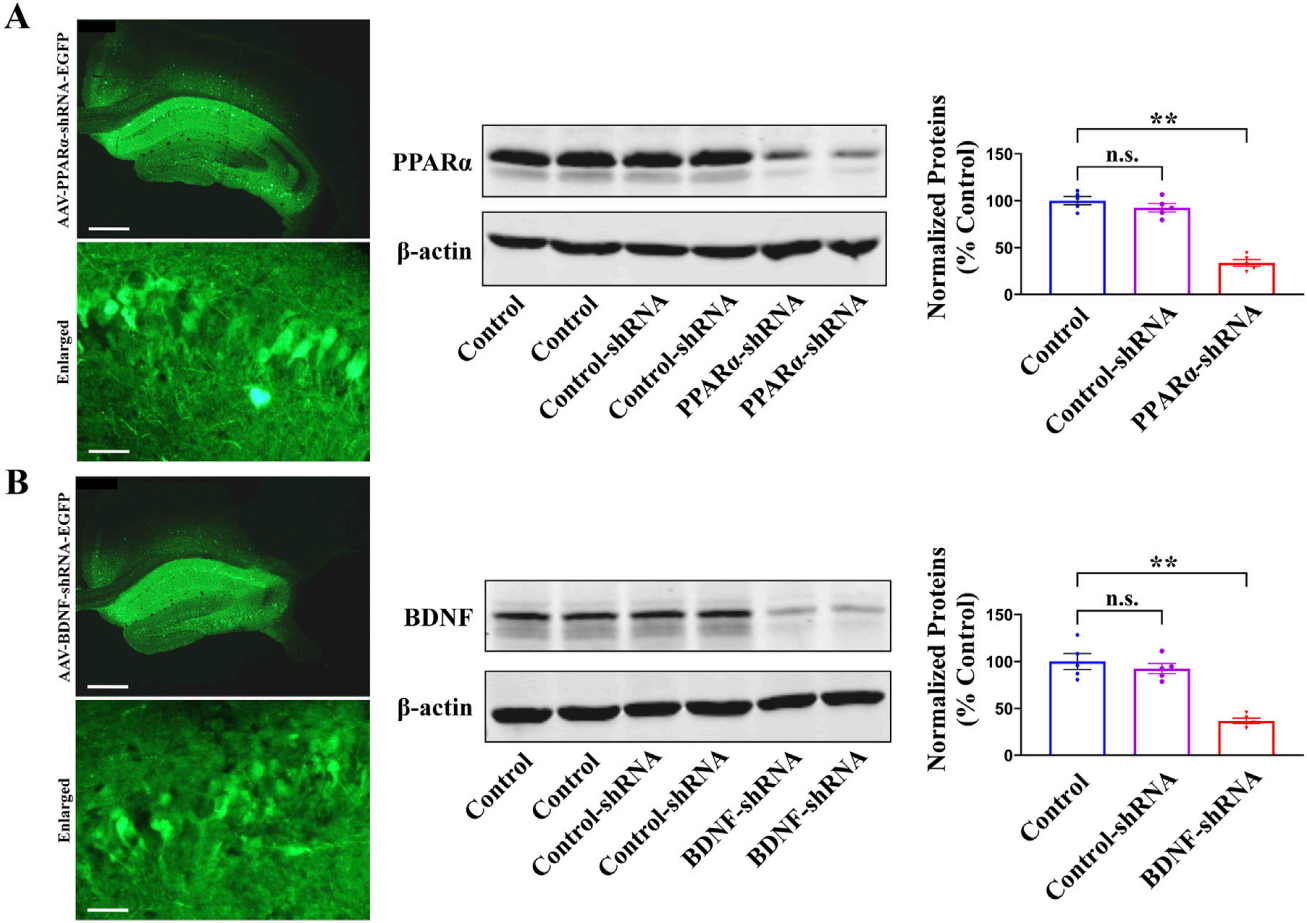
The fluorescence images of a fixed hippocampal slice expressing AAV-PPARα-shRNA-EGFP **(A)** or AAV-BDNF-shRNA-EGFP **(B)**, and the scale bars for representative and enlarged images are 400 and 50 μm, respectively. Two weeks were required for AAV-PPARα-shRNA-EGFP and AAV-BDNF-shRNA-EGFP to spread over the whole hippocampus of mice. The following western blotting results showed that the usage of PPARα-shRNA **(A)** and BDNF-shRNA **(B)** remarkably downregulated the protein expression of hippocampal PPARα and BDNF in naïve mice, respectively. All data were represented as means ± S.E.M (n = 5); **P < 0.01; n.s., no significance. The comparisons were made by one-way ANOVA followed by Tukey’s test.

Considering that pharmacological antagonists may have non-selective effects, AAV-PPARα-short hairpin RNA (shRNA)-enhanced green fluorescent protein (EGFP) and AAV-BDNF-shRNA-EGFP were respectively used to selectively knockdown the expression of hippocampal PPARα and BDNF in mice. As shown in Figure 6A, B, the silencing efficacy of PPARα-shRNA [ANOVA: F(2, 12) = 40.155, *P* < 0.01] and BDNF-shRNA [ANOVA: F(2, 12) = 37.268, *P* < 0.01] have been confirmed (n = 5, *P* < 0.01).

**FIGURE 7:**
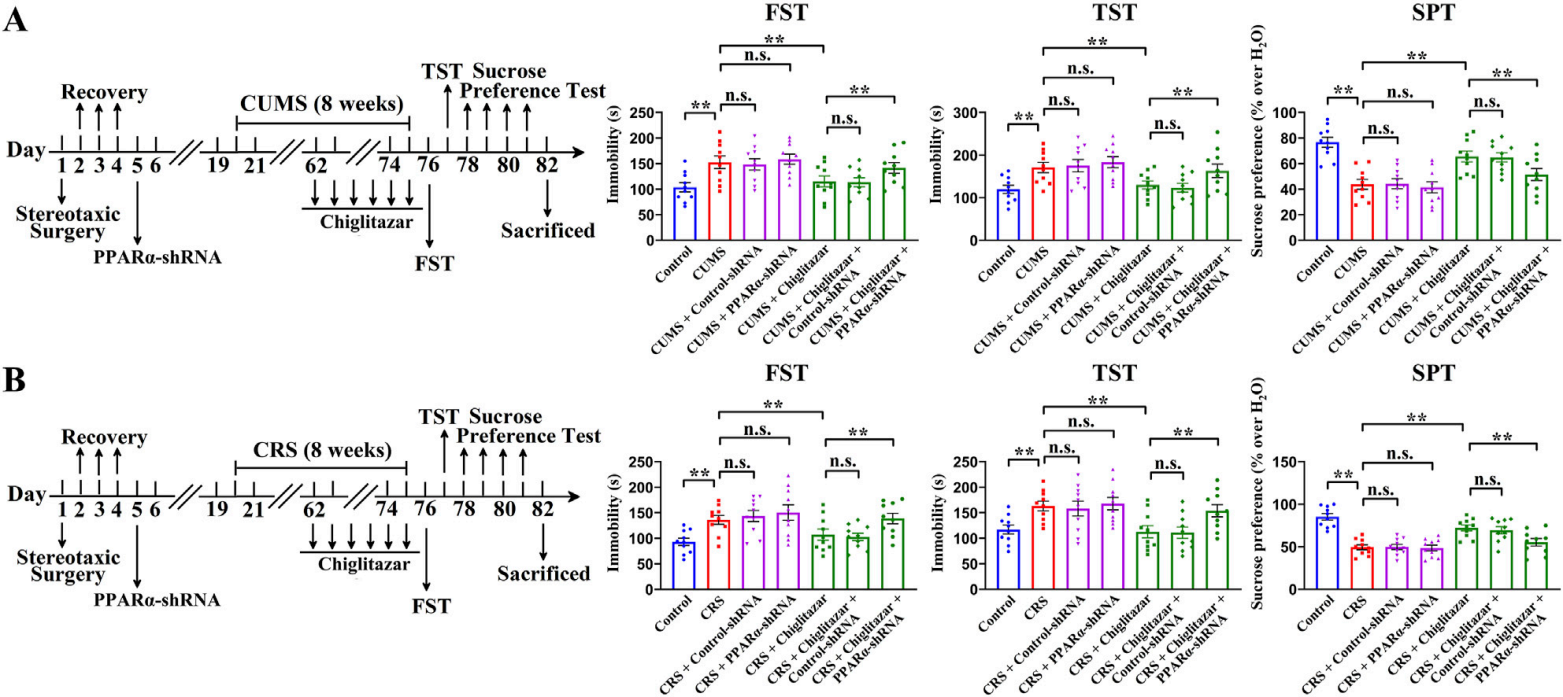
Adult C57BL/6J mice received stereotactic infusion of PPARα-shRNA or Control-shRNA first and then 8 weeks of CUMS or CRS, followed by daily injection of 10 mg/kg chiglitazar or vehicle during the last 2 weeks. **(A)** It was found that pre-treatment with PPARα-shRNA, but not Control-shRNA, significantly abolished the reversal effects of 10 mg/kg chiglitazar against the CUMS-induced increase in mice immobility in the FST and TST as well as decrease in mice sucrose preference in the SPT. A schematic timeline of the experimental procedures is provided. **(B)** It was found that pre-treatment of PPARα-shRNA, but not Control-shRNA, also notably abrogated the reversal effects of 10 mg/kg chiglitazar against the CRS-induced increase in mice immobility in the FST and TST as well as decrease in mice sucrose preference in the SPT. A schematic timeline of the experimental procedures is provided. All data were represented as means ± S.E.M (n = 10); **P < 0.01; n.s., no significance. The comparisons were made by one-way ANOVA followed by Tukey’s test.

Figure 7A shows that pre-treatment with PPARα-shRNA significantly abolished the antidepressant-like effects of chiglitazar in the CUMS model of depression, as the (CUMS + chiglitazar + PPARα-shRNA)-treated mice displayed significantly higher immobility in the FST [ANOVA: F(6, 63) = 23.846, *P* < 0.01] and TST [ANOVA: F(6, 63) = 25.141, *P* < 0.01] as well as lower sucrose preference [ANOVA: F(6, 63) = 27.596, *P* < 0.01] than both the (CUMS + chiglitazar)-treated and (CUMS + chiglitazar + Control-shRNA)-treated mice (n = 10, *P* < 0.01). Figure 7B reveals that pre-treatment of PPARα-shRNA also notably abrogated the antidepressant-like actions of chiglitazar in the CRS model of depression, as the (CRS + chiglitazar + PPARα-shRNA)-treated mice had evidently more immobility in the FST [ANOVA: F(6, 63) = 21.437, *P* < 0.01] and TST [ANOVA: F(6, 63) = 24.802, *P* < 0.01] as well as less sucrose preference [ANOVA: F(6, 63) = 26.949, *P* < 0.01] than both the (CRS + chiglitazar)-treated and (CRS + chiglitazar + Control-shRNA)-treated mice (n = 10, *P* < 0.01). The usage of Control-shRNA produced none influence on mice behaviors (n = 10).

**FIGURE 8:**
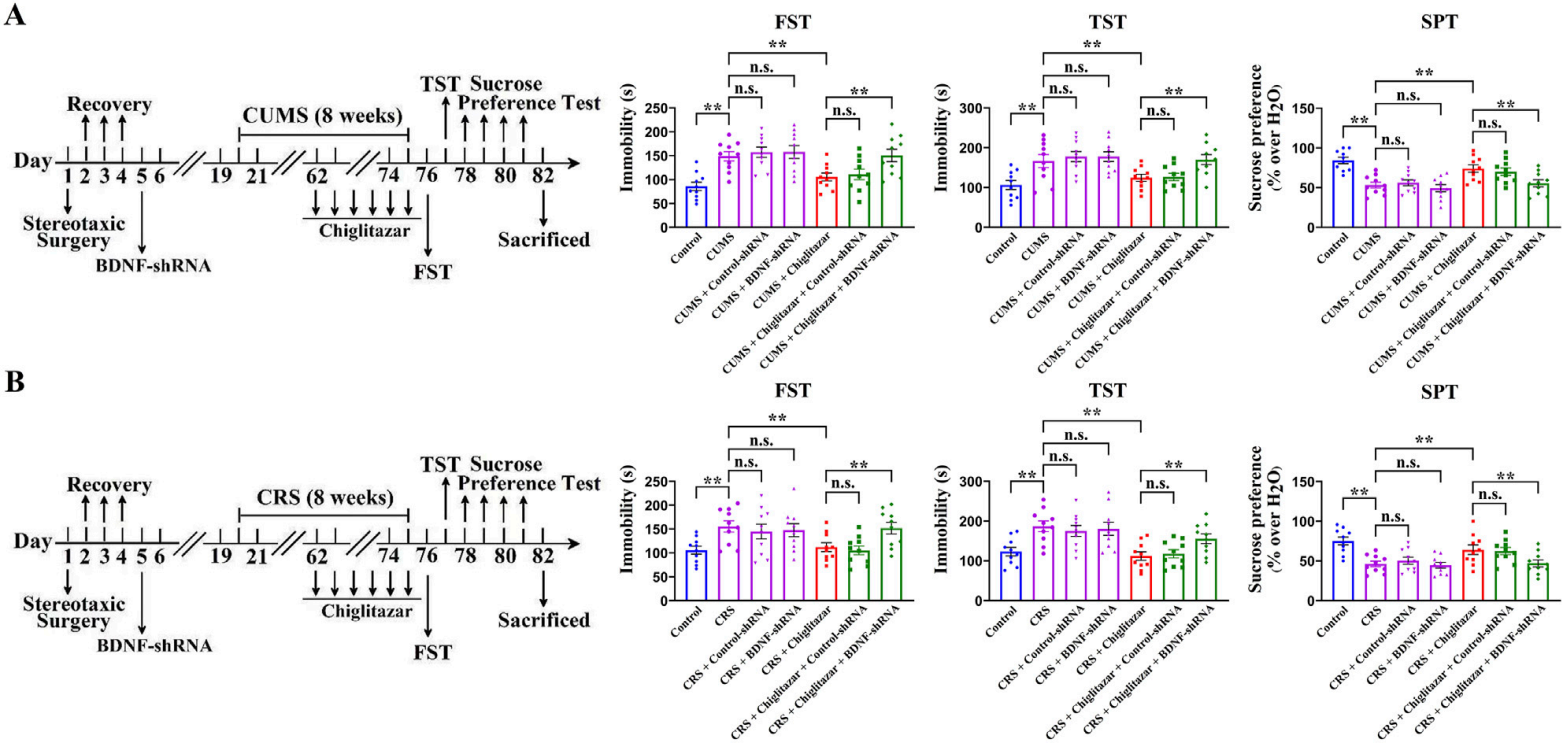
Adult C57BL/6J mice received stereotactic infusion of BDNF-shRNA or Control-shRNA first and then 8 weeks of CUMS or CRS, followed by daily injection of 10 mg/kg chiglitazar or vehicle during the last 2 weeks. **(A)** It was found that pre-treatment with BDNF-shRNA, but not Control-shRNA, significantly abolished the reversal effects of 10 mg/kg chiglitazar against the CUMS-induced increase in mice immobility in the FST and TST as well as decrease in mice sucrose preference in the SPT. A schematic timeline of the experimental procedures is provided. **(B)** It was found that pre-treatment of BDNF-shRNA, but not Control-shRNA, also notably abrogated the reversal effects of 10 mg/kg chiglitazar against the CRS-induced increase in mice immobility in the FST and TST as well as decrease in mice sucrose preference in the SPT. A schematic timeline of the experimental procedures is provided. All data were represented as means ± S.E.M (n = 10); **P < 0.01; n.s., no significance. The comparisons were made by one-way ANOVA followed by Tukey’s test.

Similarly, Figure 8A shows that pre-treatment with BDNF-shRNA significantly abolished the antidepressant-like effects of chiglitazar in the CUMS model of depression, as the (CUMS + chiglitazar + BDNF-shRNA)-treated mice displayed significantly higher immobility in the FST [ANOVA: F(6, 63) = 23.941, *P* < 0.01] and TST [ANOVA: F(6, 63) = 27.445, *P* < 0.01] as well as lower sucrose preference [ANOVA: F(6, 63) = 19.301, *P* < 0.01] than both the (CUMS + chiglitazar)-treated and (CUMS + chiglitazar + Control-shRNA)-treated mice (n = 10, *P* < 0.01). Figure 8B reveals that pre-treatment of BDNF-shRNA also notably abrogated the antidepressant-like actions of chiglitazar in the CRS model of depression, as the (CRS + chiglitazar + BDNF-shRNA)-treated mice had evidently more immobility in the FST [ANOVA: F(6, 63) = 21.162, *P* < 0.01] and TST [ANOVA: F(6, 63) = 25.349, *P* < 0.01] as well as less sucrose preference [ANOVA: F(6, 63) = 17.894, *P* < 0.01] than both the (CRS + chiglitazar)-treated and (CRS + chiglitazar + Control-shRNA)-treated mice (n = 10, *P* < 0.01).

In summary, combined with the above pharmacological results involving GW6471 and K252a, it can be found that hippocampal PPARα and BDNF signaling are required for the antidepressant-like effects of chiglitazar in mice.

## Discussion

To our knowledge, the present study is the first comprehensive *in vivo* study directly identifying that chiglitazar has beneficial effects against depression and possesses potential as a new antidepressant candidate. For mice models of depression, both CUMS and CRS were used. For behavioral assay, the FST, TST, and SPT were adopted together. CUMS is the most widely acknowledged and used rodent model in depression research and can induce several core symptoms of depression such as helplessness and anhedonia (Antoniuk et al., 2019; Sharma et al., 2024). CRS is also frequently used in depression research in recent years (Mao et al., 2022). The FST and TST are commonly employed to evaluate the helplessness behaviors of rodents, whereas the SPT is commonly performed to assess the anhedonia behaviors of rodents (Stukalin et al., 2020; Armario, 2021; Primo et al., 2023). Fluoxetine, the positive control, showed notable reversal effects against both CUMS and CRS in the FST, TST, and SPT, suggesting that our methods were reliable and effective.

Regarding the molecular mechanism underlying the antidepressant-like effects of chiglitazar found in this study, as three other pharmacological agonists for PPARα, WY14643, fenofibrate, and gemfibrozil, all have been demonstrated to produce antidepressant-like actions in mice via activating the hippocampal BDNF signaling cascade (Jiang et al., 2015; Jiang et al., 2017; Ni et al., 2018), here we went on to consider BDNF. As expected, our results involving BDNF blotting, K252a, and BDNF-shRNA together reveal that this molecule indeed underlies the antidepressant-like effects of chiglitazar in mice. Our study is the first to support a positive effect of chiglitazar on the hippocampal BDNF system and may be beneficial to further studies on the pharmacological effects and clinical use of chiglitazar. As an essential neurotrophic factor in the central nervous system, BDNF is closely implicated in the pathophysiology of not only depression but also many other neurological and psychiatric disorders such as Parkinson’s disease, Alzheimer’s disease, stroke, and schizophrenia (Björkholm and Monteggia, 2016; Amidfar et al., 2020; Han and Deng, 2020; Palasz et al., 2020; Karantali et al., 2021; Gao L. et al., 2022; Ali et al., 2024). Therefore, it would be of great significance to explore whether chiglitazar administration also produces protective effects against such disorders (Parkinson’s disease, Alzheimer’s disease, stroke, schizophrenia, etc.) in the future. It is well-known that hippocampal BDNF regulates hippocampal neurogenesis and synaptic plasticity, and depression is accompanied with not only abnormal behaviors but also dysfunction in these two physiological processes (Leal et al., 2014; Serafini et al., 2014; Liu and Nusslock, 2018; Berger et al., 2020; Tartt et al., 2022). In addition to hippocampal BDNF, BDNF in several other brain regions including the medial prefrontal cortex (mPFC) and nucleus accumbens (NAc) play important roles in the pathogenesis of depression as well (Berton et al., 2006; Xu et al., 2017; Xu et al., 2018; Chen et al., 2021). Thus, investigating the effects of chiglitazar treatment on synaptic plasticity, hippocampal neurogenesis, and BDNF in the mPFC and NAc will be part of our next plan.

We ascribed the enhancing effects of chiglitazar on hippocampal BDNF expression to hippocampal PPARα, as PPARα directly modulates BDNF biosynthesis by transcriptionally regulating CREB, the downstream signaling molecule of BDNF (Roy et al., 2013). A supporting literature comes from Roy *et al.* which demonstrated that simvastatin treatment upregulated the hippocampal BDNF expression in mice via the PPARα-mediated transcriptional activation of CREB (Roy et al., 2015). As to how PPARα activates CREB, it has been demonstrated that PPARα directly interact with CREB by binding peroxisome proliferator response elements (PPREs) (Roy et al., 2013). Chiglitazar is a pan agonist of PPARα/PPARδ/PPARγ. It should be noticed that in addition to PPARα, PPARδ and PPARγ have been reported to correlate with depression as well. For example, Liu *et al.* showed that CUMS exposure markedly reduced the hippocampal PPARδ expression in rats (Liu et al., 2017). Chen *et al.* indicated that genetic knockdown of hippocampal PPARδ caused depression-like behaviors and suppression in hippocampal neurogenesis in mice (Chen F. et al., 2019). In contrast, Ji *et al.* indicated that hippocampal PPARδ overexpression or activation repressed stress-induced depressive behaviors and enhanced hippocampal neurogenesis in mice (Ji et al., 2015). Li *et al.* further revealed that telmisartan activated hippocampal PPARδ to improve symptoms of CUMS-induced depression in mice (Li et al., 2017). Pioglitazone and rosiglitazone, two PPARγ agonists, have been demonstrated to have antidepressant effects in clinical studies (Colle et al., 2017). By analyzing these literatures, it is possible that as well as hippocampal PPARα, hippocampal PPARδ and PPARγ also participate in the antidepressant-like actions of chiglitazar in mice, and this possibility has been preliminarily ruled out by the usage of GW6471 and PPARα-shRNA in the present study. In our next plan, PPARδ inhibitors, PPARγ inhibitors, PPARδ-shRNA and PPARγ-shRNA will be also adopted.

There may be a limitation for this study, as we have used only male C57BL/6J mice, while female subjects were not included due to limited resources in our laboratory. On the other hand, the present study relies mainly on behavioral tests to assess the antidepressant-like effects of chiglitazar in mice, without detcting changes in neurotransmitters (serotonin, noradrenaline, etc.) or other depression-related biomarkers (inflammatory cytokines, oxidative stress markers, etc.). Moreover, there are some other acknowledged models of depression besides CUMS and CRS, such as the chronic social defeat stress (CSDS) model of depression (Wang W. et al., 2021). The conclusion of this study would be further confirmed if chiglitazar treatment also reverses the CSDS-induced depressive-like symptoms in rodents. These limitations or shortcomings will be solved in the future. In addition to mortality associated with suicide, patients with major depressive disorder (MDD) are more likely to develop coronary artery disease and type 2 diabetes (Li et al., 2025). Considering that chiglitazar has recently been approved in China to treat type 2 diabetes due to its moderate glucose-lowering effect, this medication may have specific advantage in treating MDD patients with type 2 diabetes in clinical practice in the future, compared to not only commonly used SSRIs and SNRIs but also other PPARα agonists such as fenofibrate and gemfibrozil. Besides, a randomized, double-blind, placebo-controlled, phase 3 trial (CMAP) in 2021 has already confirmed the safety of chiglitazar in patients with type 2 diabetes, and this can further strengthen the advantage of chiglitazar over those antidepressant candidates which have recently been reported but not been introduced into clinical trials yet (Ji et al., 2021).

Overall, the present study is the first *in vivo* comprehensive evidence showing that chiglitazar has potential of being a novel antidepressant candidate. It provides a new insight into understanding the pharmacological effects of chiglitazar and sheds light on the development of novel antidepressants with higher efficacy and fewer side effects.

## Data Availability

The original contributions presented in the study are included in the article/Supplementary Material, further inquiries can be directed to the corresponding authors.

## Abbreviations

AAV, adeno-associated virus; AKT, protein kinase B; ANOVA, analysis of variance; BDNF, brain derived neurotrophic factor; CREB, cAMP response element-binding protein; CRS, chronic restraint stress; CUMS, chronic unpredictable mild stress; ERK, extracellular regulated protein kinase; FST, forced swimming test; i.p., intraperitoneally; mPFC, medial prefrontal cortex; NAc, nucleus accumbens; PPARα, peroxisome proliferator activated receptor α; shRNA, short hairpin RNA; SNRIs, serotonin and norepinephrine reuptake inhibitors; SPT, sucrose preference test; SSRIs, selective serotonin reuptake inhibitors; TrkB, tyrosine kinase B; TST, tail suspension test.
