# Supplementary material for: Administration of chiglitazar reverses chronic stress-induced depressive-like symptoms in mice via activation of hippocampal PPARα and BDNF
Source: Front Pharmacol. 2025 Apr 28;16:1587399. doi: 10.3389/fphar.2025.1587399 (PMC12066578; doi:10.3389/fphar.2025.1587399)
Supplement: Supplementary file 1 [file DataSheet2.docx]

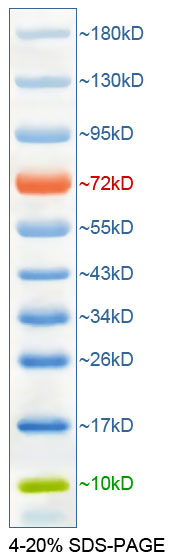


The protein marker adopted for western blotting in the present study was bought from Beyotime Biotechnology (P0068).


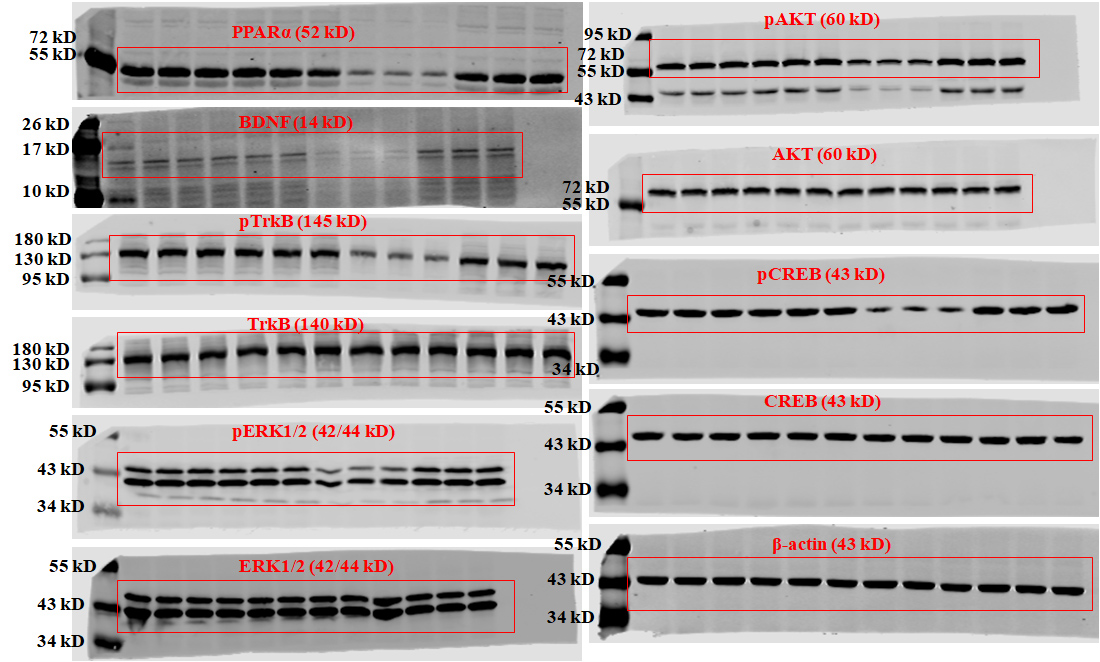


These are the original uncropped images of western blots for PPARα, BDNF, pTrkB, TrkB, pERK1/2, ERK1/2, pAKT, AKT, pCREB, CREB, and β-actin in Figure 2A.


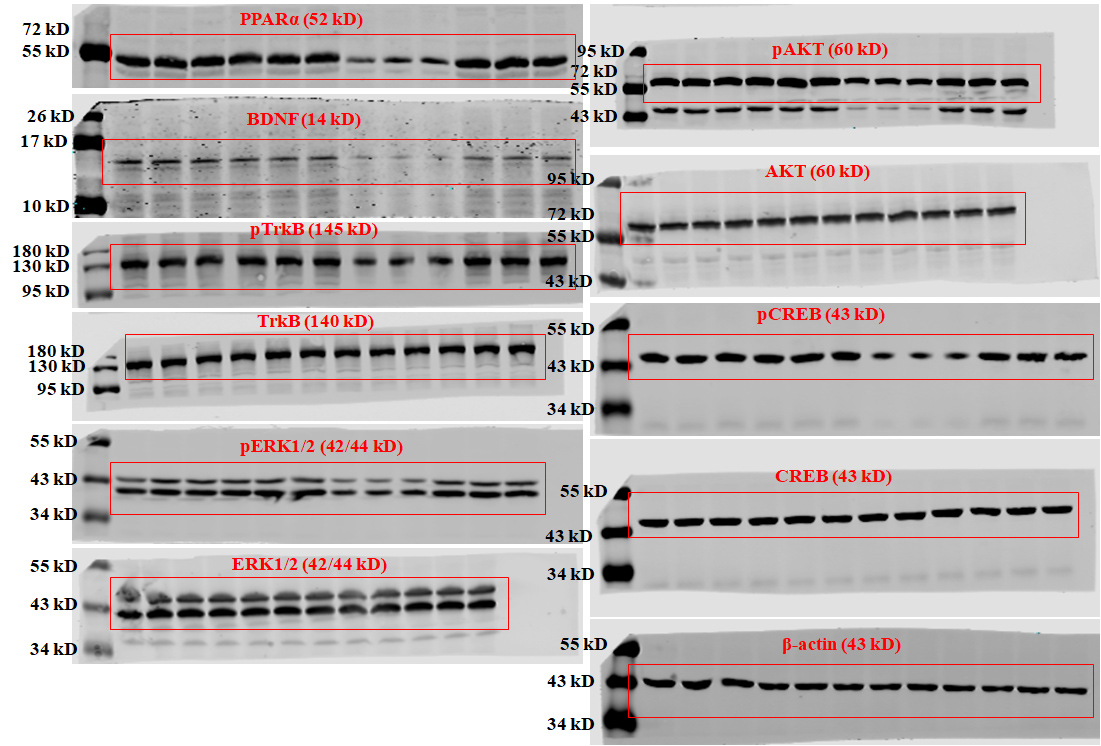


These are the original uncropped images of western blots for PPARα, BDNF, pTrkB, TrkB, pERK1/2, ERK1/2, pAKT, AKT, pCREB, CREB, and β-actin in Figure 3A.


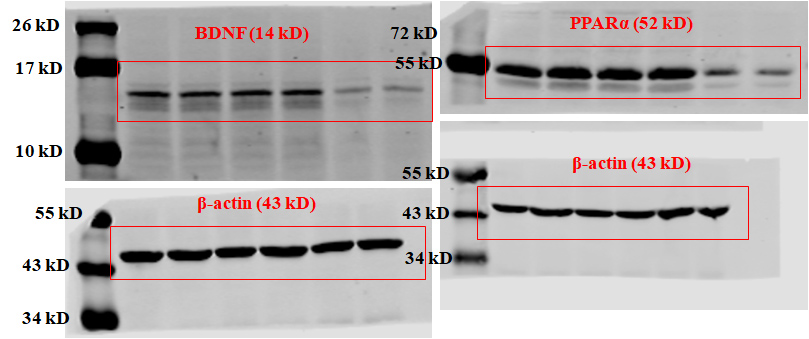


These are the original uncropped images of western blots for PPARα, BDNF, and β-actin in Figure 5A and 5B.
