# Supplementary material for: Administration of chiglitazar reverses chronic stress-induced depressive-like symptoms in mice via activation of hippocampal PPARα and BDNF
Source: Front Pharmacol. 2025 Apr 28;16:1587399. doi: 10.3389/fphar.2025.1587399 (PMC12066578; doi:10.3389/fphar.2025.1587399)
Supplement: Supplementary file 2 [file DataSheet1.docx]

**Supplemental Information**

**Supplemental Methods and Materials**

**Animals and Ethical Statements**

This study utilized 8-week-old male C57BL/6J mice (weighing 22-24 g) as experimental subjects, which were purchased from Shanghai SLAC Laboratory Animal Co., Ltd. Prior to the experiment, all mice were acclimatized in our facility for 1 week under the following housing conditions: group-housed with 5 mice per cage; 12-hour light/dark cycle (lights on from 06:00 to 18:00); ambient temperature of (24 ± 1)°C; relative humidity of (55 ± 10)%; noise levels below 50 dB; ammonia concentration below 14 mg/m³; 24-hour air circulation; and bedding replaced twice a week. These conditions were consistent with previous studies (Jiang *et al.*, 2017; Song *et al.*, 2018). Subsequently, the mice were subjected to stratified randomization according to body weight. All animal procedures adhered to the ARRIVE guidelines (Kilkenny *et al.*, 2010; McGrath & Lilley, 2015) and approved by the Institutional Animal Care and Use Committee of the Sixth People’s Hospital of Nantong (P20230916-014). All behavioral tests were conducted during the daytime (8:00 to 17:00). For in vitro studies, animals were randomly selected and euthanized at 9:00 AM using a standard method (anesthetized with carbon dioxide followed by cervical dislocation). The sample sizes (in vivo, n = 10; in vitro, n = 5) were determined by power analysis and based on previous studies (Chen *et al.*, 2019; Liu *et al.*, 2020; Wang *et al.*, 2021; Wu *et al.*, 2022; Huang *et al.*, 2023).

**Drugs**

Chiglitazar, fluoxetine (the positive control), K252a, and GW6471 were purchased from Target Mol (Boston, United States) and intraperitoneally (i.p., 10 ml/kg) injected. According to previous studies (Jiang *et al.*, 2015; Jiang *et al.*, 2017; Song *et al.*, 2018; Chen *et al.*, 2019; Liu *et al.*, 2024), the doses of these compounds were determined as following: chiglitazar (10 mg/kg), fluoxetine (20 mg/kg), K252a (25 μg/kg), and GW6471 (1 mg/kg). Normal saline containing 10% dimethyl sulfoxide and 20% Cremophor EL was utilized as the vehicle for chiglitazar, fluoxetine, K252a, and GW6471.

**Behavioral Assessments**

The protocols for the forced swim test (FST), tail suspension test (TST), sucrose preference test (SPT), and open field test (OFT) have been described in numerous previous reports (Chen *et al.*, 2019; Liu *et al.*, 2020; Wang *et al.*, 2021; Wu *et al.*, 2022; Huang *et al.*, 2023).

In the FST, each mouse was individually placed into a transparent cylindrical container provided by XinRuan Technology Co., Ltd. (Shanghai, China), with a diameter of 20 cm and a height of 45 cm. The mice were required to swim continuously for 6 min in pure water filled to a depth of 20 cm, maintained at a temperature of 24 ± 1°C. The water was replaced after each trial. During the final 4 min of the test, an investigator blinded to the animal groupings recorded the immobility time of each mouse. Immobility was defined as the mouse being completely motionless or exhibiting only minor movements necessary to maintain breathing.

In the TST, each test mouse was secured with adhesive tape 1 cm from the tip of its tail and suspended 70 cm above the ground. The test duration was 6 min, during which an investigator blinded to the animal groupings recorded the immobility time of each mouse. Immobility was defined as the state in which the mouse remained completely motionless. To prevent potential climbing behaviors during the test, transparent hollow anti-climbing tubes (1.5 cm in diameter, 4 cm in length) provided by XinRuan Technology Co., Ltd. were used.

In the SPT, each test mouse was individually housed and acclimated to two identical bottles, one containing a 1% sucrose solution and the other containing pure water, for a period of 48 h. To prevent the mice from developing a preference for a specific position, the locations of the two bottles were switched every 12 h. Following this, the mice underwent 18 h of food and water deprivation, after which the pre-weighed bottles were returned, and the mice were allowed to drink freely for 3 h (with the bottle positions switched every 1 h). After the experiment, the bottles were weighed again. The sucrose preference index for each mouse was calculated as a percentage using the formula: 100 × volume of sucrose consumed / total volume consumed.

In the OFT, a square arena (100 × 100 × 50 cm) with white walls and flooring was utilized. The arena was positioned in a dimly lit room illuminated by a 50 W red bulb. The floor of the arena was marked with black lines to form a grid of 25 identical squares (20 × 20 cm). Mice were individually allowed to freely explore the arena for 5 min, and an experimenter blinded to group assignments documented the number of squares traversed by each animal. Between trials, the floor was thoroughly cleaned to eliminate residual odors.

**Adeno-Associated Virus (AAV)-Mediated Gene Knockdown**

This experimental method was adapted from previous reports (Song *et al.*, 2018; Liu *et al.*, 2020; Wu *et al.*, 2022; Huang *et al.*, 2023) with appropriate modifications. During the experiment, mice were anesthetized with 0.5% pentobarbital sodium and secured in a stereotaxic apparatus (Stoelting, USA). First, the scalp of each mouse was incised, and the skull was cleaned and exposed using 75% ethanol and 1% H_2_O_2_. Subsequently, 5-μl microsyringes were used to inject AAV-peroxisome proliferator-activated receptor α (PPARα)-short hairpin RNA (shRNA)-enhanced green fluorescent protein (EGFP), AAV-brain derived neurotrophic factor (BDNF)-shRNA-EGFP, or AAV-Control-shRNA-EGFP. After drilling a small hole in the mouse skull, the microsyringe was precisely positioned at the following coordinates: AP = -2.3 mm, ML = ±1.6 mm, DV = +1.8 mm (Wu *et al.*, 2022; Huang *et al.*, 2023). The AAV constructs were bilaterally injected into the hippocampus at a rate of 0.5 μl per minute (1.5 μl per side). After injection, the microsyringe was left in place for 5 min to prevent viral reflux. Finally, the incision was sutured, and the mice were allowed to recover for 3 d before proceeding with further experiments. The AAV-Control-shRNA-EGFP, AAV-PPARα-shRNA-EGFP, and AAV-BDNF-shRNA-EGFP used in the experiment were provided by Dr. Bo Jiang, and their preparation methods have been described in detail in previous studies (Song *et al.*, 2018; Jiang *et al.*, 2019). The sequences for PPARα-shRNA, BDNF-shRNA, and Control-shRNA were 5’-AGAAATTCTTACCTGTGAA-3’, 5’-TGAGCGTGTGTGACAGTATTA-3’, and 5’-TTCTCCGAACGTGTCACGT-3’, respectively (Song *et al.*, 2018; Chen *et al.*, 2019; Huang *et al.*, 2023). They were diluted to 2 × 10^10^ TU/ml before use. Two weeks were required for PPARα-shRNA and BDNF-shRNA to produce gene knockdown effects.

**Western Blotting**

During the experiment, the hippocampal tissues were rapidly and directly dissected from the brain of each test mouse immediately after sacrifice. Subsequently, the tissues were homogenized using NP-40 lysis buffer containing 1 mM PMSF, protease inhibitors, and phosphatase inhibitors. After homogenization, the protein supernatant was extracted from the lysate by centrifugation (12,000 × g, 4°C, 15 min) and subjected to denaturation (95°C, 5 min). Protein concentration was determined using a commercial BCA kit. The subsequent experimental steps were carried out according to the following commonly used protocol (Jiang *et al.*, 2017; Song *et al.*, 2018; Chen *et al.*, 2019; Jiang *et al.*, 2019; Wang *et al.*, 2021; Wu *et al.*, 2022): First, 30 μg of protein samples were separated using 10/12% SDS-PAGE. Next, the proteins were transferred to nitrocellulose membranes. Then, the membranes were blocked with 5% non-fat dry milk in TBST for 2 h at room temperature (RT). After blocking, the membranes were washed three times with TBST at RT, 15 min each. Subsequently, the membranes were incubated with diluted primary antibodies overnight at 4°C. Following primary antibody incubation, the membranes were washed three times with TBST at RT, 15 min each. Then, the membranes were incubated with diluted secondary antibodies for 2 h at RT. After secondary antibody incubation, the membranes were washed three times with TBST at RT, 15 min each. Finally, the membranes were scanned.

Primary antibodies against PPARα (1:500; Abcam, Bristol, UK), BDNF (1:500; Abcam), tyrosine receptor kinase B (TrkB; 1:1000; Abcam), phospho-TrkB-Tyr^516^ (pTrkB; 1:500; Thermo Fisher, Waltham, USA), extracellular regulated protein kinase 1/2 (ERK1/2, 1:1000; Cell Signaling, Danvers, USA), phospho-ERK1/2-Thr^202^/Tyr^204^ (pERK1/2; 1: 1000; Cell Signaling), protein kinase B (AKT, 1: 1000; Cell Signaling), phospho-AKT-Ser^473^ (pAKT, 1: 500; Cell Signaling), cAMP response element-binding protein (CREB; 1:1000; Cell Signaling), phospho-CREB-Ser^133^ (pCREB; 1:500; Cell Signaling), and β-actin (1:5000; Cell Signaling) were used. IR-Dye® 680RD-labelled donkey anti-rabbit secondary antibody (1:10000; Licor, Lincoln, USA) was also used. The Odyssey® Infrared Imaging system and ImageJ software were adopted to perform blot scanning and analyzing respectively.

**Data and Statistical Analysis**

The experimental data are presented as mean ± standard error of the mean (S.E.M.). For statistical analysis, SPSS 22.0 software (SPSS Inc., Chicago, USA) was used, with one-way analysis of variance (ANOVA) combined with Tukey's test and two-way ANOVA combined with Bonferroni's test. The statistical significance level was set at *P* < 0.05 (two-tailed).

**References**

Chen, C., Shen, J.H., Xu, H., Chen, P., Chen, F., Guan, Y.X., Jiang, B. & Wu, Z.H. (2019) Hippocampal PPARα is involved in the antidepressant-like effects of venlafaxine in mice. *Brain Res Bull*, **153**, 171-180.

Huang, J., Fan, H., Chen, Y.M., Wang, C.N., Guan, W., Li, W.Y., Shi, T.S., Chen, W.J., Zhu, B.L., Liu, J.F. & Jiang, B. (2023) The salt-inducible kinases inhibitor HG-9-91-01 exhibits antidepressant-like actions in mice exposed to chronic unpredictable mild stress. *Neuropharmacology*, **227**, 109437.

Jiang, B., Huang, C., Zhu, Q., Tong, L.J. & Zhang, W. (2015) WY14643 produces anti-depressant-like effects in mice via the BDNF signaling pathway. *Psychopharmacology (Berl)*, **232**, 1629-1642.

Jiang, B., Wang, H., Wang, J.L., Wang, Y.J., Zhu, Q., Wang, C.N., Song, L., Gao, T.T., Wang, Y., Meng, G.L., Wu, F., Ling, Y., Zhang, W. & Li, J.X. (2019) Hippocampal Salt-Inducible Kinase 2 Plays a Role in Depression via the CREB-Regulated Transcription Coactivator 1-cAMP Response Element Binding-Brain-Derived Neurotrophic Factor Pathway. *Biol Psychiatry*, **85**, 650-666.

Jiang, B., Wang, Y.J., Wang, H., Song, L., Huang, C., Zhu, Q., Wu, F. & Zhang, W. (2017) Antidepressant-like effects of fenofibrate in mice via the hippocampal brain-derived neurotrophic factor signalling pathway. *Br J Pharmacol*, **174**, 177-194.

Kilkenny, C., Browne, W., Cuthill, I.C., Emerson, M. & Altman, D.G. (2010) Animal research: reporting in vivo experiments: the ARRIVE guidelines. *Br J Pharmacol*, **160**, 1577-1579.

Liu, L., Sun, W., Tang, X., Zhen, D., Guan, C., Fu, S. & Liu, J. (2024) Chiglitazar attenuates high-fat diet-induced nonalcoholic fatty liver disease by modulating multiple pathways in mice. *Mol Cell Endocrinol*, **593**, 112337.

Liu, Y., Tang, W., Ji, C., Gu, J., Chen, Y., Huang, J., Zhao, X., Sun, Y., Wang, C., Guan, W., Liu, J. & Jiang, B. (2020) The Selective SIK2 Inhibitor ARN-3236 Produces Strong Antidepressant-Like Efficacy in Mice via the Hippocampal CRTC1-CREB-BDNF Pathway. *Front Pharmacol*, **11**, 624429.

McGrath, J.C. & Lilley, E. (2015) Implementing guidelines on reporting research using animals (ARRIVE etc.): new requirements for publication in BJP. *Br J Pharmacol*, **172**, 3189-3193.

Song, L., Wang, H., Wang, Y.J., Wang, J.L., Zhu, Q., Wu, F., Zhang, W. & Jiang, B. (2018) Hippocampal PPARα is a novel therapeutic target for depression and mediates the antidepressant actions of fluoxetine in mice. *Br J Pharmacol*, **175**, 2968-2987.

Wang, Y., Gu, J.H., Liu, L., Liu, Y., Tang, W.Q., Ji, C.H., Guan, W., Zhao, X.Y., Sun, Y.F., Xu, D.W. & Jiang, B. (2021) Hippocampal PPARα Plays a Role in the Pharmacological Mechanism of Vortioxetine, a Multimodal-Acting Antidepressant. *Front Pharmacol*, **12**, 673221.

Wu, Z.H., Fan, H., Gao, S.Y., Jin, Y.F., Cheng, C., Jiang, B. & Shen, J. (2022) Antidepressant-like activity of oroxylin A in mice models of depression: A behavioral and neurobiological characterization. *Front Pharmacol*, **13**, 921553.
